# Supplementary material for: Efficiencies of O-MBR and A/O-MBR for Organic Matter Removal from and Trihalomethane Formation Potential Reduction in Domestic Wastewater
Source: Membranes (Basel). 2022 Aug 2;12(8):761. doi: 10.3390/membranes12080761 (PMC9416499; doi:10.3390/membranes12080761)
Supplement: Supplementary file 1 [file membranes-12-00761-s001.zip › membranes-1807919-supplementary.pdf]

Supplementary Materials

Efficiencies of O-MBR and A/O-MBR for Organic Matter Removal from and Trihalomethane Formation Potential Reduction in Domestic Wastewater

Sornsiri Sriboonnak[1], Aegkapan Yanun[1], Phacharapol Induvesa[2], Chayakorn Pumas[3,4], Kritsana Duangjan[5], Pharkphum Rakruam[1], Saoharit Nitayavardhana[1], Prattakorn Sittisom[1] and Aunnop Wongrueng[1,3],*

[1] Department of Environmental Engineering, Faculty of Engineering, Chiang Mai University, Chiang Mai 50200, Thailand; sornsiri_sri@cmu.ac.th (S.S.); benentaneer41@gmail.com (A.Y.); pharkphum@eng.cmu.ac.th (P.R.); saoharit@eng.cmu.ac.th (S.N.); prattakorn.s@cmu.ac.th (P.S.); aunnop@eng.cmu.ac.th (A.W.)

[2] Bodhivijjalaya College, Srinakharinwirot University, Nakhon Nayok 26120, Thailand; phacharapol@g.swu.ac.th (P.I.)

[3] Research Center in Bioresources for Agriculture, Industry and Medicine, Chiang Mai University, Chiang Mai 50200, Thailand

[4] Department of Biology, Faculty of Science, Chiang Mai University, Chiang Mai 50200, Thailand; chayakorn.pumas@gmail.com (C.P.)

[5] Science and Technology Research Institute, Chiang Mai University, Chiang Mai 50200, Thailand; kritsana.du@gmail.com (K.D.)

* Correspondence: aunnop@eng.cmu.ac.th; Tel.: +66 53 94 4101-3, Fax. +66 53 94 4105

Effect of pH, alkalinity, dissolved oxygen, and ratio of MLVSS to MLSS

The effect of A/O-MBR and O-MBR systems on pH values are shown in Table S1 and Figures S1 and S2. Raw wastewater influent showed an insignificant change in pH value for both systems at different SRTs ($p>0.05$). The average pH value of wastewater that entered the system in the experiments under SRT=10 days, SRT=20 days, and SRT=infinity was equal to 7.51±0.1. The pH values of wastewater in the oxic tanks of A/O-MBR and O-MBR systems were in the range of 7.12-7.31 under all SRTs. This reveals that they are suitable for microbial growth and that nitrification reactions were not inhibited [19]. In the case of the anoxic tank of the A/O-MBR system, the pH values of wastewater were 7.22-7.31. This suggested that certain pH values could prevent the inhibition of the nitrification reaction in the anoxic tank. The optimal range of pH values for the nitrification reaction have been reported to be between 7.0 and 8.0 [20]. The pH values of permeate from A/O-MBR and O-MBR systems were in the range of 7.18-7.28. It was found that those values met the effluent pH standard of 5.0-9.0 [1].

The alkalinities of raw wastewater and wastewater in A/O-MBR and O-MBR systems are reported in Table S2 and Figure S3. Raw wastewater influent showed alkalinity values for both systems in the range of 123.1-137.1 mg/L as $CaCO_3$ under SRT=10 days, SRT=20 days, and SRT=infinity, which was sufficient for nitrification reactions. Alkalinity values of wastewater in anoxic tanks of the A/O-MBR system were in the range of 164.9-172.1 mg/L as $CaCO_3$ under all SRTs. The alkalinity values of wastewater in oxic tanks of the A/O-MBR system were in the range of 170.9-174.8 mg/L as $CaCO_3$ under all SRTs. The alkalinity values of wastewater in oxic tanks of the O-MBR system were in the range of 98.8-128.9 mg/L as $CaCO_3$ under all SRTs. The alkalinity was within the permissible limits for the nitrification process. The alkalinity in the oxic tanks of the O-MBR system decreased due to its use in the nitrification reaction. Microorganisms need sufficient alkalinity to operate as electron receptors [21-22].

The dissolved oxygen (DO) level in wastewater after treatment with A/O-MBR and O-MBR systems are reported in Table S3 and DO levels during the experiment at different SRTs in the A/O-MBR system (System1) and O-MBR system (System2) are shown in Figure S4. The DO values of wastewater in the anoxic tanks of the A/O-MBR system were in the range of 0.4-0.5 mg/L under all SRTs. These values were ideal for an anaerobic tank [23]. The DO values of wastewater in the oxic tanks of the A/O-MBR system were in the range of 6.5-7.3 mg/L under all SRTs. The DO values of wastewater in the oxic tanks of the O-MBR system were in the range of 6.3-7.5 mg/L under all SRTs. It has been suggested that the DO value in an aeration tank should be at least 2.0 mg/L [24].

The ratio of MLVSS to MLSS (MLVSS/MLSS) of the wastewater in the A/O-MBR and O-MBR systems is reported in Table S4 and Figure S5. In the A/O-MBR system, the MLVSS/MLSS values of the wastewater in the anoxic tanks were in the range of 0.53-0.61 mg/L under all SRTs. In the A/O-MBR system's oxic tank, the MLVSS/MLSS values of the wastewater were in the range of 0.62-0.73 under all SRTs. The MLVSS/MLSS values of the wastewater in the anoxic

tanks were slightly lower than those obtained from the wastewater in the oxic tanks. This may explain how the anoxic tank microbes used organic carbon from outside and collected them in their cells rather than using them for cell synthesis [25]. This may consequently have contributed to the low MLVSS and MLVSS/MLSS values. Regarding the O-MBR system, the MLVSS/MLSS values of wastewater in the oxic tank were in the range of 0.66-0.71 under all SRTs.

Table S1. effects of A/O-MBR and O-MBR systems on pH value.

SRT (days)	pH					
		A/O-MBR (System 1)			O-MBR (System 2)	
	Raw wastewater	Wastewater in Anoxic tank	Wastewater in Oxic tank	Permeate	Wastewater in Oxic tank	Permeate
10	7.53±0.15	7.22±0.18	7.26±0.14	7.26±0.13	7.12±0.14	7.18±0.19
20	7.48±0.14	7.29±0.13	7.31±0.09	7.26±0.075	7.15±0.13	7.24±0.15
Infinity	7.52±0.18	7.31±0.08	7.30±0.09	7.28±0.071	7.20±0.08	7.22±0.17
p-value	0.055	0.000	0.009	0.351	0.001	0.044

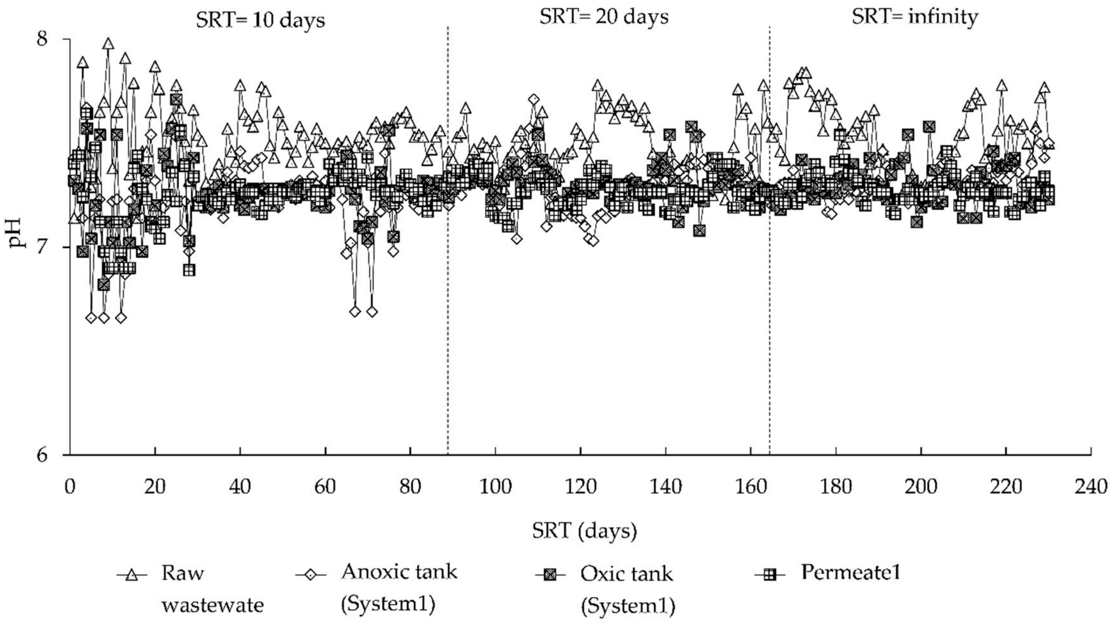

Figure S1. pH during experiment at different SRTs in the A/O-MBR system (System 1).

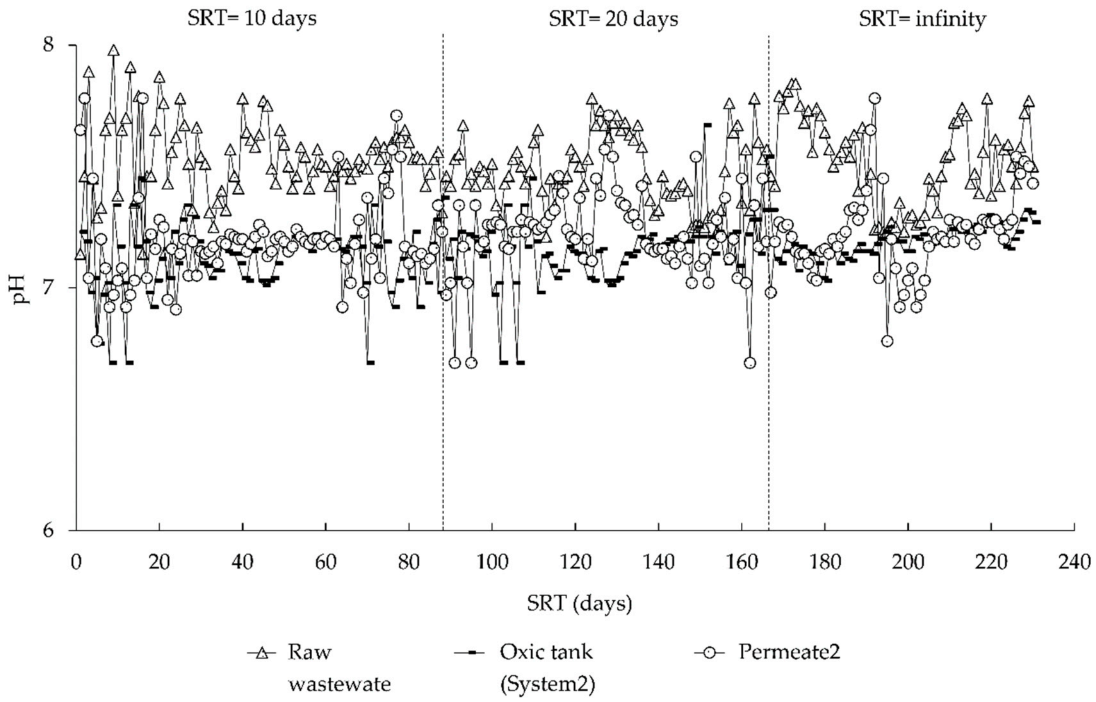

Figure S2. pH during experiment at different SRTs in the O-MBR system (System 2).

Table S2. alkalinities of raw wastewater and wastewater in the A/O-MBR and O-MBR systems.

SRT (days)	Alkalinity (mg/L as $CaCO_3$)			
		A/O-MBR (System 1)		O-MBR (System 2)
	Raw wastewater	Wastewater in Anoxic tank	Wastewater in Oxic tank	Wastewater in Oxic tank
10	137.1±18.4	164.9±11.3	170.9±4.4	128.9±22.4
20	123.1±19.8	169.6±5.6	172.1±7.2	106.7±11.3
Infinity	128.8±20.5	172.1±5.3	174.8±5.8	98.8±10.9
p-value	0.070	0.019	0.135	0.000

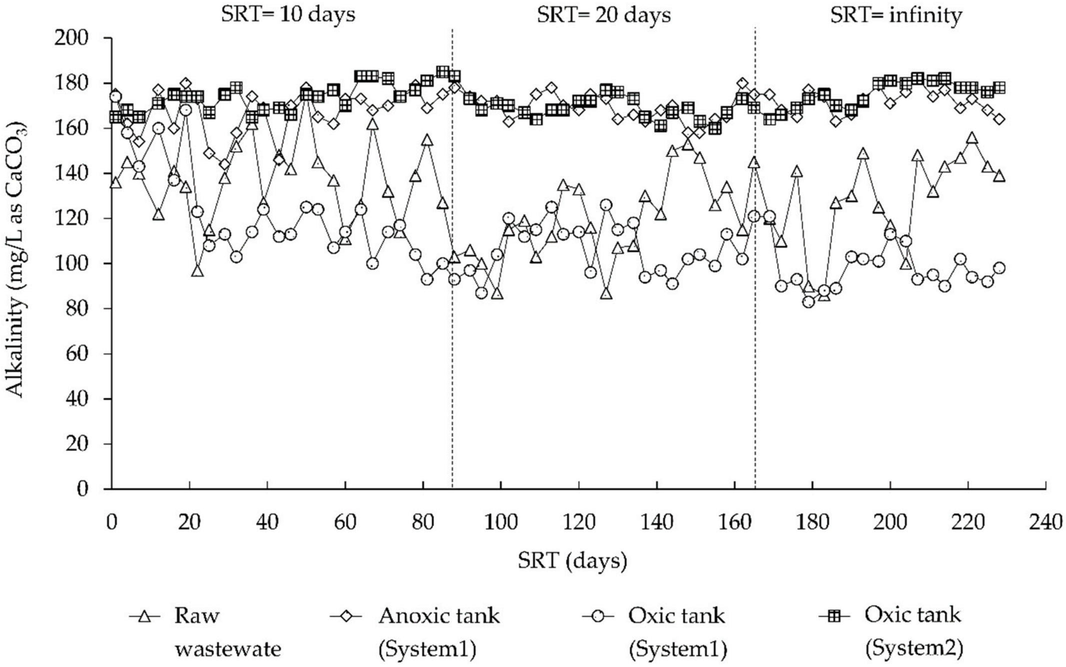

Figure S3. alkalinity during experiment at different SRTs in the A/O-MBR system (System1) and O-MBR system (System 2).

Table S3. dissolved oxygen in raw wastewater and wastewater in the A/O-MBR and O-MBR systems.

SRT (days)	DO (mg/L)		
	A/O-MBR (System 1)		O-MBR (System 2)
	Wastewater in Anoxic tank	Wastewater in Oxic tank	Wastewater in Oxic tank
10	0.5±0.1	6.9±0.4	7.1±0.3
20	0.4±0.0	6.5±0.2	6.3±0.4
Infinity	0.4±0.0	7.3±0.2	7.5±0.1
p-value	0.011	0.000	0.000

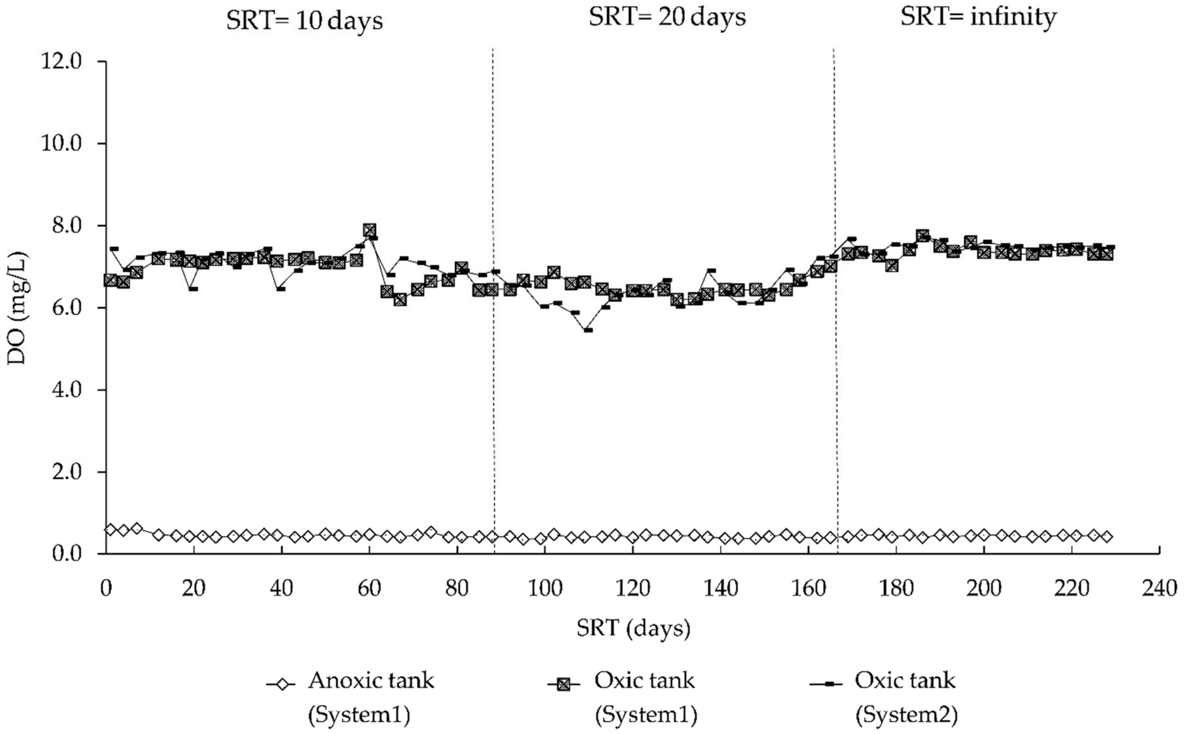

Figure S4. DO during experiment at different SRTs in the A/O-MBR system (System 1) and O-MBR system (System 2).

Table S4. MLVSS/MLSS of raw wastewater and wastewater in the A/O-MBR and O-MBR systems.

SRT (days)	MLVSS/MLSS		
	A/O-MBR (System 1)		O-MBR (System 2)
	Wastewater in Anoxic tank	Wastewater in Oxic tank	Wastewater in Oxic tank
10	0.53±0.07	0.62±0.10	0.67±0.08
20	0.57±0.05	0.73±0.04	0.71±0.04
Infinity	0.61±0.05	0.66±0.03	0.66±0.03
p-value	0.000	0.000	0.006

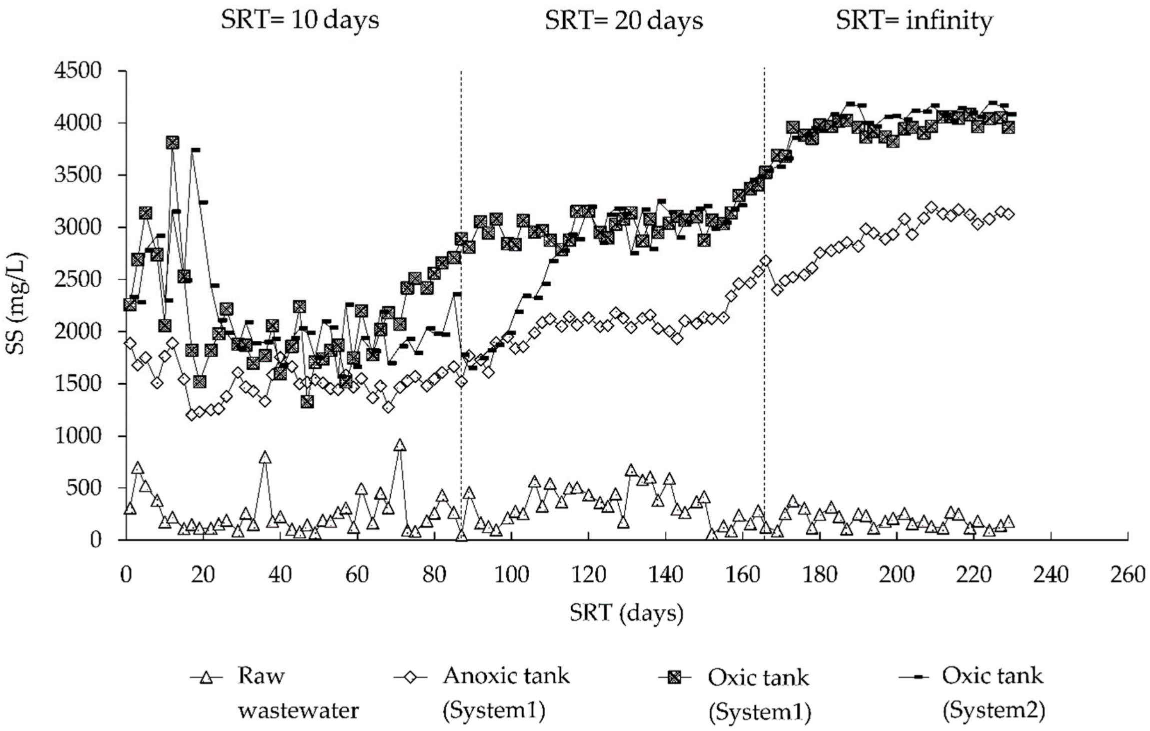

Figure S5. SS during experiment at different SRTs.

Table S5. efficiencies of A/O-MBR and O-MBR systems on total Kjeldahl nitrogen (TKN) removal.

SRT (days)	total Kjeldahl nitrogen (mg/L)		
	Raw wastewater	Permeate from A/O-MBR (System 1)	Permeate from O-MBR (System 2)
10	33.6±33.1	2.3±0.2	4.2±0.7
20	31.4±4.6	1.5±0.3	1.5±0.4
Infinity	32.3±3.1	1.2±0.1	1.2±0.2
p-value	0.052	0.000	0.000

Table S6. efficiencies of A/O-MBR and O-MBR systems on nitrite (NO_2^-) removal.

SRT (days)	nitrite (mg/L)		
	Raw wastewater	Permeate from A/O-MBR (System 1)	Permeate from O-MBR (System 2)
10	1.1±0.4	1.5±1.3	2.9±2.6
20	0.2±0.2	0.2±0.2	0.3±0.3
Infinity	0.0±0.0	0.2±0.1	0.2±0.1
p-value	0.000	0.000	0.005

Table S7. efficiencies of A/O-MBR and O-MBR systems on nitrate (NO_3^-) removal.

SRT (days)	nitrate (mg/L)		
	Raw wastewater	Permeate from A/O-MBR (System 1)	Permeate from O-MBR (System 2)
10	0.8±0.3	18.2±1.8	21.9±2.5
20	0.1±0.1	11.0±0.9	22.1±2.7
Infinity	0.1±0.1	7.5±0.7	20.0±1.9
p-value	0.000	0.000	0.002

References

1. Domestic Wastewater. Available online: https://www.pcd.go.th/ (accessed on 2 May 2022).
19. Amatya, I.; Kansakar, B.; Tare, V.; Fiksdal, L., Role of pH on biological Nitrification Process. *Journal of the Institute of Engineering* **2011,** *8*.
20. Ovez, B., Batch biological denitrification using Arundo donax, Glycyrrhiza glabra, and Gracilaria verrucosa as carbon source. *Process Biochemistry* **2006,** *41*, 1289-1295.
21. Ding, Y.; Guo, Z.; Ma, B.; Wang, F.; You, H.; Mei, J.; Hou, X.; Liang, Z.; Li, Z.; Jin, C., The Influence of Different Operation Conditions on the Treatment of Mariculture Wastewater by the Combined System of Anoxic Filter and Membrane Bioreactor. *Membranes* **2021,** *11*, 729.
22. Fu, Z.; Yang, F.; An, Y.; Xue, Y., Simultaneous nitrification and denitrification coupled with phosphorus removal in an modified anoxic/oxic-membrane bioreactor (A/O-MBR). *Biochemical Engineering Journal* **2009,** *43* (2), 191-196.
23. Painter, H. A.; Loveless, J. E., Effect of temperature and pH value on the growth-rate constants of nitrifying bacteria in the activated-sludge process. *Water Research* **1983,** *17*, 237-248.
24. Metcalf and Eddy. *Wastewater Engineering Treatment: Treatment, Disposal and Reuse,* 3rd Edition, ISBN 0-07-041690-7, McGraw-Hill, Singapore, 1991.
25. Do, K.U.; Chu, X.Q., Chapter 5 - Performances of membrane bioreactor technology for treating domestic wastewater operated at different sludge retention time. In *Development in Wastewater Treatment Research and Processes*, Shah, M.; Rodriguez-Couto, S.; Biswas, J., Eds. Elsevier: 2022; pp 107-122.
